# Prognosis Implication of N-Terminal Pro-B-Type Natriuretic Peptide in Adult Patients With Acute Myocarditis

**DOI:** 10.3389/fcvm.2022.839763

**Published:** 2022-03-30

**Authors:** Yan Zhao, Naqiang Lyu, Wei Zhang, Huiqiong Tan, Qi Jin, Aimin Dang

**Affiliations:** ^1^State Key Laboratory of Cardiovascular Disease, Department of Special Care Center, National Center for Cardiovascular Diseases, Fuwai Hospital, Chinese Academy of Medical Sciences and Peking Union Medical College, Beijing, China; ^2^Emergency and Critical Care Center, National Center for Cardiovascular Diseases, Fuwai Hospital, Chinese Academy of Medical Sciences and Peking Union Medical College, Beijing, China; ^3^Center for Pulmonary Vascular Diseases, National Center for Cardiovascular Diseases, Fuwai Hospital, Chinese Academy of Medical Sciences and Peking Union Medical College, Beijing, China; ^4^Department of Cardiology, Zhongshan Hospital, Shanghai Institute of Cardiovascular Diseases, Fudan University, Shanghai, China

**Keywords:** NT-proBNP, prognosis, biomarker, risk stratification, acute myocarditis

## Abstract

**Background:**

The aim of this study is to investigate the role of N-terminal pro-B-type natriuretic peptide (NT-proBNP) in assessing the poor outcomes of adult patients with acute myocarditis.

**Methods:**

A total of 170 adult patients with available NT-proBNP information were included in the study. They were grouped according to quartiles of NT-proBNP concentrations at admission. Baseline and follow-up information was collected. Thirty-day major adverse cardiac events (MACE) were death and heart transplantation. Long-term MACE included all-cause death, heart transplantation, re-hospitalization due to heart failure, sustained ventricular arrhythmia, and myocarditis relapse.

**Results:**

During a median follow-up of 3.8 years, patients in the highest NT-proBNP quartile suffered from the highest risk both of 30-day and long-term MACE (*P* < 0.001 by log-rank test). Multivariate analysis showed that apart from left ventricular ejection fraction (LVEF), an increased baseline NT-proBNP > 3,549 pg/mL (hazard ratio 3.535, 95% CI 1.316–9.499, *P* = 0.012) and NT-proBNP > 7,204 pg/mL (hazard ratio 22.261, 95% CI 1.976–250.723, *P* = 0.012) was independent predictor of long-term and 30-day MACE, respectively.

**Conclusions:**

Higher baseline NT-proBNP level was an independent predictor of poor outcomes in adult patients with acute myocarditis. Therefore, NT-proBNP may serve as a useful biomarker for risk stratification in acute myocarditis patients.

## Introduction

Acute myocarditis, an inflammatory disease of myocardium, can have various clinical presentations. The prognosis of patients with myocarditis also varies according to distinct etiology. Most of the patients present with mild symptoms and recover completely, however, pathological data show myocarditis in 8.6% of cases of sudden death in young adults (1), while up to 30% of myocarditis proved by biopsy might develop to dilated cardiomyopathy, which is the most common disease requiring heart transplantation (2). Uncommonly, patients with hemodynamically unstable myocarditis, which is called fulminant myocarditis, face an extremely high risk of death due to sudden onset cardiogenic shock, ventricular arrhythmias, or multiorgan failure and have a dismal prognosis (3).

Therefore, it is crucial to identify high-risk patients as early as possible to provide an optimized treatment strategy in order to improve their outcomes. Although endomyocardial biopsy (EMB) remains to be gold standard for diagnosis and prognosis of myocarditis, it is not performed widely and routinely (4). Previous studies identified prolonged QRS or QTc interval, decreased LVEF, as well as specific location and pattern of late gadolinium enhancement (LGE) in cardiac magnetic resonance (CMR) as predictors for poor prognosis in patients with acute myocarditis (5–10). However, imaging tools such as echocardiography and CMR fail to reflect subtle changes that precede cardiac dysfunction and tissue fibrosis and could hardly be monitored continuously. Hence there is a need to find out a biomarker with prognostic value for adverse outcomes in acute myocarditis patients.

Natriuretic peptides are established biomarkers for the diagnosis and prognosis evaluation of various cardiovascular conditions including acute or chronic heart failure and coronary heart disease (11–13). A series of recent studies reported that N-terminal pro-B-type natriuretic peptide (NT-proBNP) levels may be a useful predictor of mortality in patients with coronavirus disease 2019 (COVID-19) related myocarditis (14–16). In the present study, we hypothesized that NT-proBNP could independently predict poor prognosis of adult patients with acute myocarditis. We tested the hypothesis with complete data of NT-proBNP from a cohort of 170 adult patients with acute myocarditis.

## Methods

### Ethics Statement

This study complied with the ethical guidelines of the Declaration of Helsinki and China's regulations and guidelines on good clinical practice and was approved by the ethics committees of Fuwai Hospital (no. 2021-1470).

### Study Design and Participants

**Figure 1:**
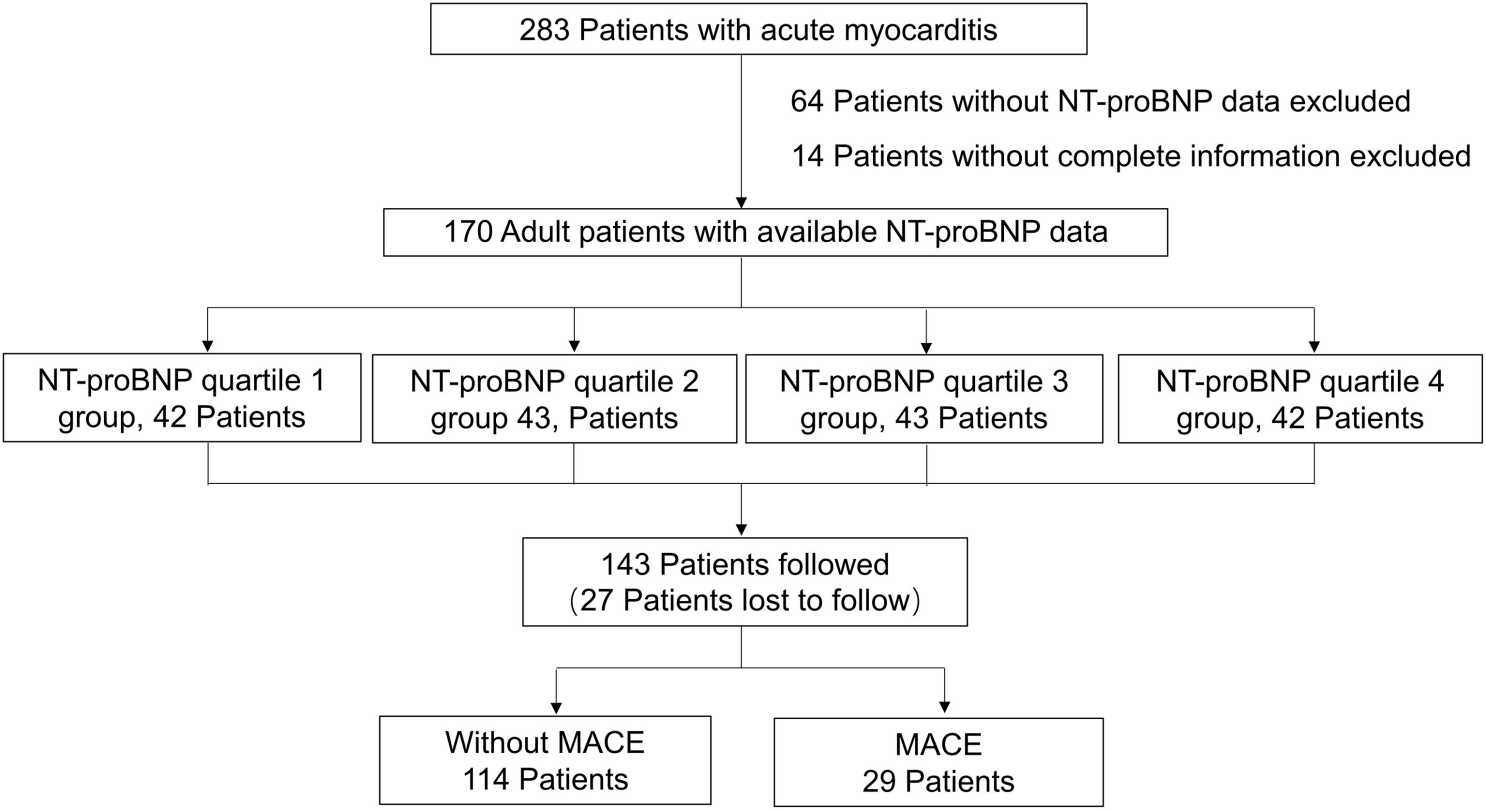
The flow chart of the enrollment of 170 adult patients with acute myocarditis from the overall population with acute myocarditis.

This single-center, retrospective, observational study was performed at Fuwai Hospital (National Center of Cardiovascular Diseases, Beijing, China). Two hundred and forty eight adult patients were clinically diagnosed with acute myocarditis from August 2006 to March 2020. Among these patients, 170 patients with complete clinical information and NT-proBNP data were selected. The electronic medical records of the patients were reviewed by trained attendings. Clinical data were collected and analyzed, including demographics, medical history, physical examination, laboratory tests, treatment measures, and outcomes. All patients had no history of ischemic or hemorrhagic stroke, renal or liver dysfunction, chronic obstructive pulmonary disease, or thyroid disease. The diagnosis of acute myocarditis was clinically based on Caforio et al. (2), we included patients with presenting symptoms or signs of two or more of the following five criteria: (1) Clinical symptoms (within 3 months): acute chest pain, dyspnea, syncope, heart failure, palpitation, or aborted cardiac death, unexplained cardiogenic shock; (2) electrocardiography (ECG) or Holter features; (3) Elevated myocardium injured markers: troponin I (TnI); (4) Echocardiography findings: functional and structural abnormalities; (5) CMR findings: consistent with myocardial inflammation, if presenting with two or more of the Lake-Louise criteria (17), namely edema, or hyperemia, and/or late gadolinium enhancement. If EMB or pathology of the hearts available after heart transplantation was consistent with the revised Dallas criteria (18), the diagnosis of myocarditis was definite. The exclusion criteria included: (1) evidence of coronary stenosis ≥ 50%; (2) other pre-existing cardiovascular disease including valvular heart disease, hypertensive heart disease, congenital heart disease or cardiomyopathy. Participants were divided into four groups according to quartiles of NT-proBNP levels: quartile 1 (<94.37 pg/mL, *n* = 42), quartile 2 group (94.37–425.47 pg/mL, *n* = 43), quartile 3 (425.47–4557.00 pg/mL, *n* = 43), and quartile 4 (>4557.00 pg/mL, *n* = 42). Figure 1 shows the flow chart of enrollment.

During hospitalization, all patients were treated based on the recommended management for myocarditis (2). Stable patients with injured left ventricular function received recommended heart failure treatment. Patients with severe or cardiogenic shock were treated with inotropes and mechanic circulatory support (MCS). MCS included intra-aortic balloon pump (IABP), venous-arterial extracorporeal membrane oxygenation (va-ECMO), or combination of IABP and va-ECMO.

### N-Terminal Pro-B-Type Natriuretic Peptide Tests

Blood samples were obtained from 1 to 3 days after admission. The blood samples were collected into tubes with EDTA-anticoagulant and centrifuged. Serum NT-proBNP was directly measured in the Laboratory of Fuwai Hospital using a commercial electrochemiluminescence assay (Roche Diagnostics GmbH, Mannheim, Germany). The reference range for plasma NT-proBNP in our laboratory was <125 pg/mL.

### Follow-Up and Outcomes

Outcome data were obtained by telephone interview, reviewing electronic medical records, or out-patient visit. All events were checked and confirmed by an independent group of trained, clinical physicians. Thirty day major adverse cardiac events (MACE) were defined as death and heart transplantation within 30 days after admission. Long-term MACE included: (1) all-cause death; (2) heart transplantation; (3) heart failure requiring hospitalization; (4) recorded sustained ventricular arrhythmia (>30s); (5) myocarditis relapse.

### Statistical Analysis

Continuous variables are presented as mean ± SD or as median (Q1-Q3). Differences in baseline participant characteristics were assessed by analysis of variance or Mann-Whitney *U* test for continuous variables and Pearson χ^2^ test or Fisher's exact test for categorical variables. The correlation between NT-proBNP and other parameters were assessed by Pearson and Spearman's correlation analysis. Univariate and multivariate Cox proportional hazards analysis was performed to determine the factors predicting MACE. The covariables included in the multivariate Cox analysis was selected according to the following reasons: the variables that were significant in univariate analysis, or the ones were reported to be associated with NT-proBNP levels and MACE (age, BMI, QRS duration > 120 ms, QTc interval > 440 ms, creatinine, LVEF <50%, etc.). Survival curves were generated by the Kaplan-Meier method and compared with the log-rank test. The ability of NT-proBNP for predicting MACE was evaluated by the receiver operating characteristic (ROC) curve analysis and quantified by the area under the ROC curve (AUC), in which a value of 1.0 indicates perfect ability and a value of 0.5 indicates no ability. All analyses were two tailed, and *P*-values <0.05 were considered statistically significant. Analyses were performed with IBM SPSS statistics software version 26.0. The Kaplan-Meier curves were made with GraphPad Prism software version 5.0 and the ROC curves were made with MedCalc software version 19.0.

## Results

### Patients Population and Clinical Presentation

The main baseline characteristics of the study population are reported in Supplementary Table 1. One hundred and seventy patients with available NT-proBNP data were included in the analysis. One hundred fourteen patients were diagnosed based on EMB or CMR, while 56 patients without EMB or CMR were diagnosed mainly according to clinical presentations, ECGs, laboratory tests and echocardiography. The baseline characteristics of the patients with or without EMB or CMR are shown in Supplementary Table 2, and there was no significant difference in NT-proBNP levels between the two groups. The population was divided according to quartiles of NT-proBNP levels (Table 1). Patients in quartile 4 were the oldest and had the highest percentage of female. No significant differences were found in the prevalence of comorbidities among the four groups. Chest pain was less in patients with lower NT-proBNP levels, while dyspnea was more frequent in patients with higher NT-proBNP, and the prevalence of syncope was of no significant difference among the four groups. Patients with higher NT-proBNP had significantly higher heart rate and lower systolic blood pressure. Patients with highest NT-proBNP levels had the most prevalence of arrythmia, including sinus tachycardia, sustained ventricular arrythmias, and bundle-branch block, as well as the longest QRS and QTc intervals. However, the prevalence of complete atrioventricular block and supraventricular tachycardia was not significantly different among the four groups. Patients with higher NT-proBNP levels presented with higher C response protein level, higher troponin I level, and worse renal function. They also had a larger left atrium, lower left ventricular ejection fraction (LVEF), and almost 70% of the patients in the highest quartile group had LVEF <50%. Treatment differed across the four groups with the most invasive life support strategies for patients with the highest NT-proBNP levels. In terms of medication, aldosterone antagonists and corticosteroids were most used in patients in the highest quartile, and no significant difference was found in the use of β-blocker and ACE-I/ARB among the participants.

**Table 1:** Baseline characteristics of the study population grouped by the quartiles of NT-proBNP.

	**Q1**	**Q2**	**Q3**	**Q4**	***P*-Value**
	**(<94.37 pg/mL,** ***n* = 42)**	**(94.37–425.47 pg/mL,** ***n* = 43)**	**(425.47–4557.00 pg/mL,** ***n* = 43)**	**(> 4557.00 pg/mL,** ***n* = 42)**	
**Demographics**					
Age (years, Q1–Q3)	27 (22–36)	26 (22–33)	34 (28–46)	36 (30–53)	<0.001
Male, *n* (%)	30 (71.4)	34 (79.1)	27 (62.8)	21 (50.0)	0.032
BMI (kg/m^2^)	23.0 ± 3.6	24.4 ± 3.9	25.3 ± 4.6	23.2 ± 4.1	0.029
**Comorbidities and NYHA class**					
Hypertension, *n* (%)	1 (2.4)	3 (7.0)	5 (11.6)	4 (9.5)	0.415
Diabetes mellitus, *n* (%)	0 (0)	2 (4.7)	0 (0)	3 (7.1)	0.132
Dyslipidemia, *n* (%)	5 (11.9)	1 (2.3)	3 (7.0)	6 (14.3)	0.213
NYHA III or IV (%)	0 (0)	13 (30.2)	24 (55.8)	36 (85.7)	<0.001
**Clinical presentation**, ***n*** **(%)**				
Chest pain	19 (45.2)	19 (44.2)	15 (34.9)	8 (19.0)	0.044
Dyspnea	8 (19.0)	11 (25.6)	23 (53.5)	28 (66.7)	<0.001
Syncope	5 (11.9)	6 (14.0)	6 (14.0)	7 (16.7)	0.941
**Vital signs at admission**					
Systolic blood pressure (mmHg)	117 ± 13	115 ± 16	107 ± 17	102 ± 17	<0.001
Diastolic blood pressure (mmHg)	68 ± 10	69 ± 13	68 ± 9	66 ± 12	0.670
Heart rate (beats/minute)	74 ± 13	80 ± 16	89 ± 24	97 ± 24	<0.001
**Electrocardiogram at admission**					
Normal, *n* (%)	26 (61.9)	10 (23.3)	10 (23.3)	1 (2.4)	<0.001
QRS interval (ms)	94 ± 22	103 ± 32	96 ± 26	115 ± 34	0.003
QTc interval (ms)	428 ± 46	436 ± 48	440 ± 42	463 ± 45	0.003
QRS interval >120 ms, *n* (%)	2 (4.8)	9 (20.9)	6 (14.0)	13 (31.0)	0.014
QTc interval >440 ms, *n* (%)	10 (25.0)	16 (42.1)	21 (51.2)	31 (75.6)	<0.001
**Arrhythmia**, ***n*** **(%)**					
Sinus tachycardia	1 (2.4)	6 (14.0)	15 (34.9)	22 (52.4)	<0.001
Supraventricular tachycardia	1 (2.4)	1 (2.3)	3 (7.0)	6 (14.3)	0.084
Sustained VT/VF	1 (2.4)	2 (4.7)	3 (7.0)	8 (19.0)	0.026
complete AVB	4 (9.5)	7 (16.3)	6 (14.0)	7 (16.7)	0.772
Bundle-branch block	2 (4.8)	10 (23.3)	7 (16.3)	13 (31.0)	0.017
**Laboratory tests at admission**					
WBC (×10^9^/L, Q1–Q3)	6.5 (5.6–8.0)	7.4 (6.5–11.1)	9.0 (6.7–11.8)	10.6 (7.0–14.5)	<0.001
CRP (mg/L, Q1–Q3)	4.4 (2.1–11.6)	13.1 (4.9–56.3)	18.0 (7.0–61.5)	28.7 (15.6–115.3)	<0.001
Creatinine (μmol/L, Q1–Q3)	75.6 (64.3–86.7)	79.0 (66.0–86.1)	82.0 (65.0–102.0)	101.3 (78.8–129.6)	<0.001
Troponin I (ng/mL, Q1–Q3)	0.65 (0.1–3.7)	2.6 (1.0–5.7)	2.7 (0.46–6.46)	2.6 (0.3–12.2)	0.014
NT-proBNP (pg/mL, Q1–Q3)	54.4(34.6–67.1)	249.0 (160.0–326.7)	1417.0 (702.2–2432.6)	9079.8 (6907.8–24143.3)	<0.001
Peak NT-proBNP (pg/mL, Q1–Q3)	57.8 (41.6–83.8)	270.3 (162.0–352.1)	1692.0 (823.0–3395.0)	16902.2(9257.4–35398.0)	<0.001
**Echocardiography at admission**					
LA (mm)	32 ± 4	33 ± 6	35 ± 6	35 ± 6	0.001
LVEDD (mm)	47 ± 5	50 ± 9	49 ± 6	51 ± 8	0.036
IVS (mm)	9 ± 2	10 ± 2	9 ± 2	10 ± 2	0.046
RV (mm)	21 ± 2	22 ± 6	21 ± 3	23 ± 5	0.094
LVEF (%)	64 ± 4	57 ± 10	48 ± 14	42 ± 13	<0.001
LVEF <50%, *n* (%)	0 (0)	6 (14.0)	25 (58.1)	29 (69.0)	<0.001
Coronary angiography or CT angiography performed, *n* (%)	34 (81.0)	31 (72.1)	32 (74.4)	29 (69.0)	0.642
No evidence of CAD, *n* (%)	34 (100)	31 (100)	32 (100)	29 (100)	-
MRI performed, *n* (%)	25 (59.5)	23 (53.5)	35 (81.4)	26 (61.9)	0.043
**Medications**					
β-Blockers, *n* (%)	30 (71.4)	30 (69.8)	34 (79.1)	33 (78.6)	0.669
ACE-I or ARB, *n* (%)	20 (47.6)	19 (44.2)	22 (51.2)	18 (42.9)	0.870
Aldosterone antagonists, n (%)	1 (2.4)	9 (20.9)	16 (37.2)	21 (50.0)	<0.001
Corticosteroids, *n* (%)	5 (7.8)	11 (17.2)	20 (31.3)	28 (43.8)	<0.001
**Life support treatment**					
MCS, *n* (%)	0 (0)	2 (4.7)	7 (16.3)	15 (35.7)	<0.001
Ventilator, *n* (%)	0 (0)	0 (0)	4 (9.3)	11 (26.2)	<0.001
Temporary pacing, *n* (%)	4 (9.5)	6 (14.0)	5 (11.6)	5 (11.9)	0.940

*BMI, Body mass index; VT, ventricular tachycardia; VF, ventricular fibrillation; AVB, atrioventricular block; WBC, white blood cell; CRP, C-reactive protein; NT-proBNP, N-terminal pro-B-type natriuretic peptide; LA, left atrium; LVEDD, left ventricular end-diastolic diameter; IVS, intraventricular septum; RV, right ventricular diameter; LVEF, left ventricular ejection fraction; CAD, coronary atherosclerosis disease; ACE-I, angiotensin-converting enzyme-inhibitor; ARB, angiotensin receptor blocker; MCS, mechanic circulatory support*.

### Etiology of Acute Myocarditis

Twenty four patients (14.1%) accepted EMB. Histopathology and Immunopathology Findings Showed Lymphocyte Myocarditis in 13 Patients (54.2%), Giant Cell Myocarditis in 3 Patients (12.5%), Eosinophilic Myocarditis in 2 Patients (8.3%), and no Typical Myocarditis Characteristics in the Other 6 Patients (25.0%). No Patient Was Diagnosed With COVID-19 After Clinical Evaluation and Screening of Virus Nucleic Acid by Nasopharyngeal or Oropharyngeal Swabs.

### Correlation Analysis of NT-ProBNP Levels With Echocardiographic Parameters and Other Parameters

The serum concentrations of NT-proBNP were mediumly and negatively correlated with LVEF (Rho = −0.616; *P* < 0.001). However, they were only slightly correlated with the left ventricular end-diastolic diameter (Rho = 0.164; *P* = 0.033) and left atrium diameter (Rho = 0.259; *P* = 0.001). Otherwise, NT-proBNP levels were mildly correlated with age (Rho = 0.361; *P* < 0.001), C reactive protein levels (Rho = 0.444; *P* < 0.001), and creatinine levels (Rho = 0.358; *P* < 0.001). They were slightly correlated with troponin I levels (Rho = 0.195; *P* = 0.011) and were not significantly related with BMI (Rho = 0.004; *P* = 0.960).

### ROC Curve Analysis and Predictive Value for MACE

**Figure 2:**
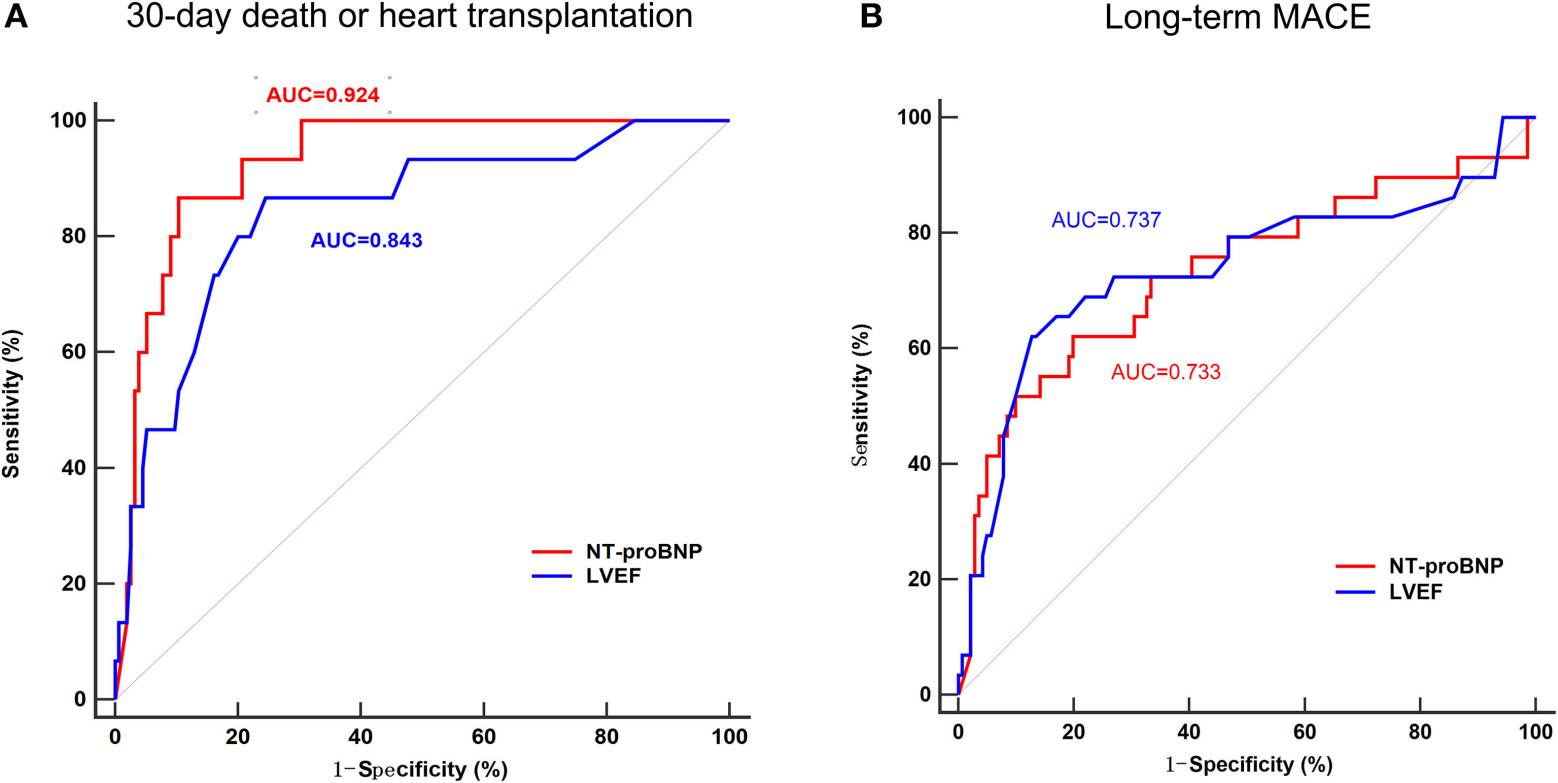
Receiver operating characteristic (ROC) curve of the ability of NT-proBNP and LVEF to predict 30-day death or heart transplantation **(A)** and long-term MACE **(B)** in patients with acute myocarditis. In predicting 30-day death or heart transplantation, the area under the curve (AUC) for NT-proBNP was 0.929, with 86.67% sensitivity and 89.68% specificity, while the AUC for LVEF was 0.843, with 86.67% sensitivity and 75.48% specificity. In predicting long-term MACE, the AUC for NT-proBNP was 0.733, with 62.07% sensitivity and 80.14% specificity, while the AUC for LVEF was 0.737, with 62.07% sensitivity and 87.23% specificity. MACE, major adverse cardiac events; NT-proBNP, N-terminal pro-B-type natriuretic peptide; LVEF, left ventricular ventricle ejection fraction.

To assess the predictive value of NT-proBNP in patients with acute myocarditis, and to compare with LVEF, the established strong predictor, ROC curves for LVEF and NT-proBNP were compared. In predicting 30-day death or heart transplantation, the sensitivity and specificity of NT-proBNP were 86.67 and 89.68%, respectively (AUC = 0.924, optimal cut-off value: 7,204 pg/mL), while the sensitivity and specificity of LVEF were 86.67 and 75.48%, respectively (AUC = 0.843, optimal cut-off value: 45%) (Figure 2A). In predicting long-term MACE, the sensitivity and specificity of NT-proBNP were 62.07 and 80.14%, respectively (AUC = 0.733, optimal cut-off value: 3,549 pg/mL). The sensitivity and specificity of LVEF to predict long-term MACE was 62.07 and 87.23%, respectively (AUC = 0.737, optimal cut-off value: 38%) (Figure 2B). ROC curves showed that the predictive values of troponin I were lower than NT-proBNP for both of 30-day (AUC = 0.616, optimal cut-off value: 15.846 ng/mL) and long-term MACE (AUC = 0.509, optimal cut-off value: 0.315 ng/mL) (Supplementary Figure 2).

### Prognostic Value of the NT-ProBNP Levels in Acute Myocarditis

After a follow-up of 3.8 ± 3.2 years, long-term MACE happened in 29 patients (17.1%) including 11 deaths (6.5%), 4 heart transplantations (2.4%), 8 heart failure hospitalizations (4.7%), 1 sustained ventricular arrhythmia (0.6%), and 5 recurrent myocarditis (2.9%). 30-day MACE occurred in 15 patients (8.8%) including 11 deaths (6.5%) and 4 heart transplantations (2.4%). There were 19 patients (11.2%) lost to follow-up, who had lower baseline NT-proBNP levels and higher LVEF at admission (Supplementary Table 3).

**Figure 3:**
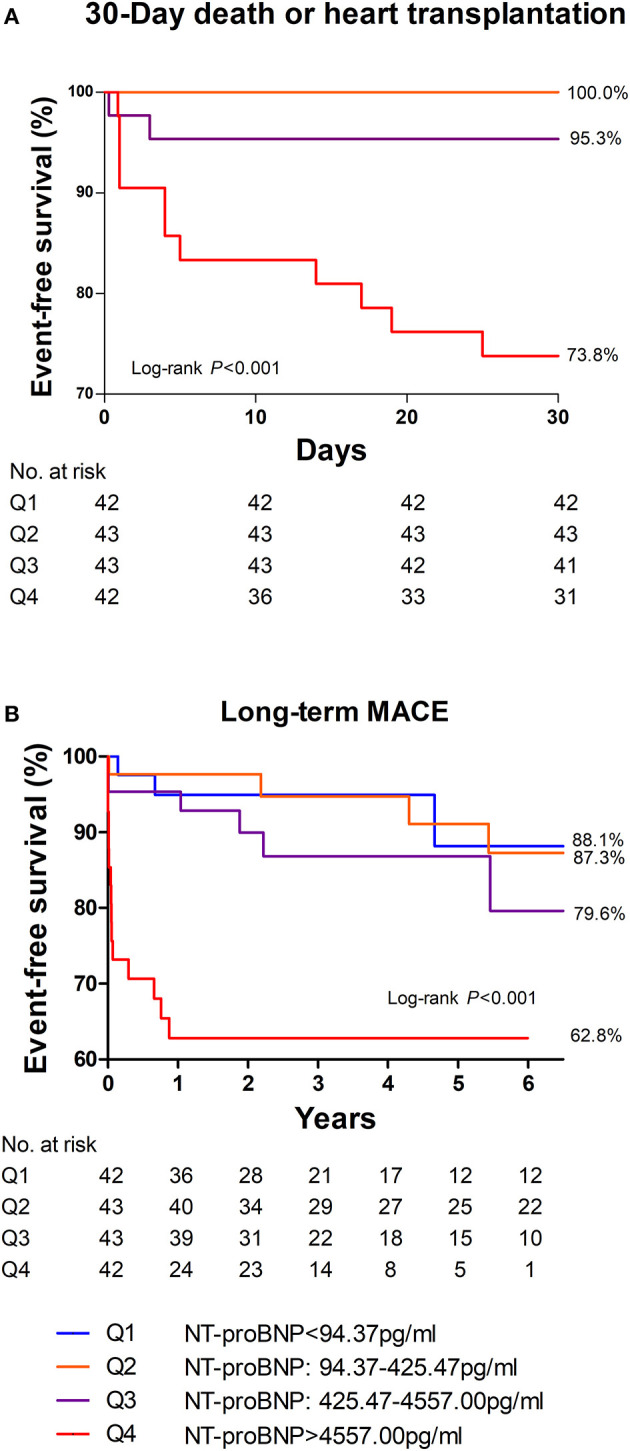
30-day **(A)** and long-term **(B)** MACE-free survival among different quartiles of NT-proBNP in patients with acute myocarditis. Patients in the highest quartile of NT-proBNP had the most MACE in 30 days as well as in the long-term. Thirty day MACE included deaths and heart transplantation within 30 days after admission. Long-term MACE included deaths, heart transplantations, re-hospitalization for heart failure, and sustained ventricular arrhythmias (>30s).

**Figure 4:**
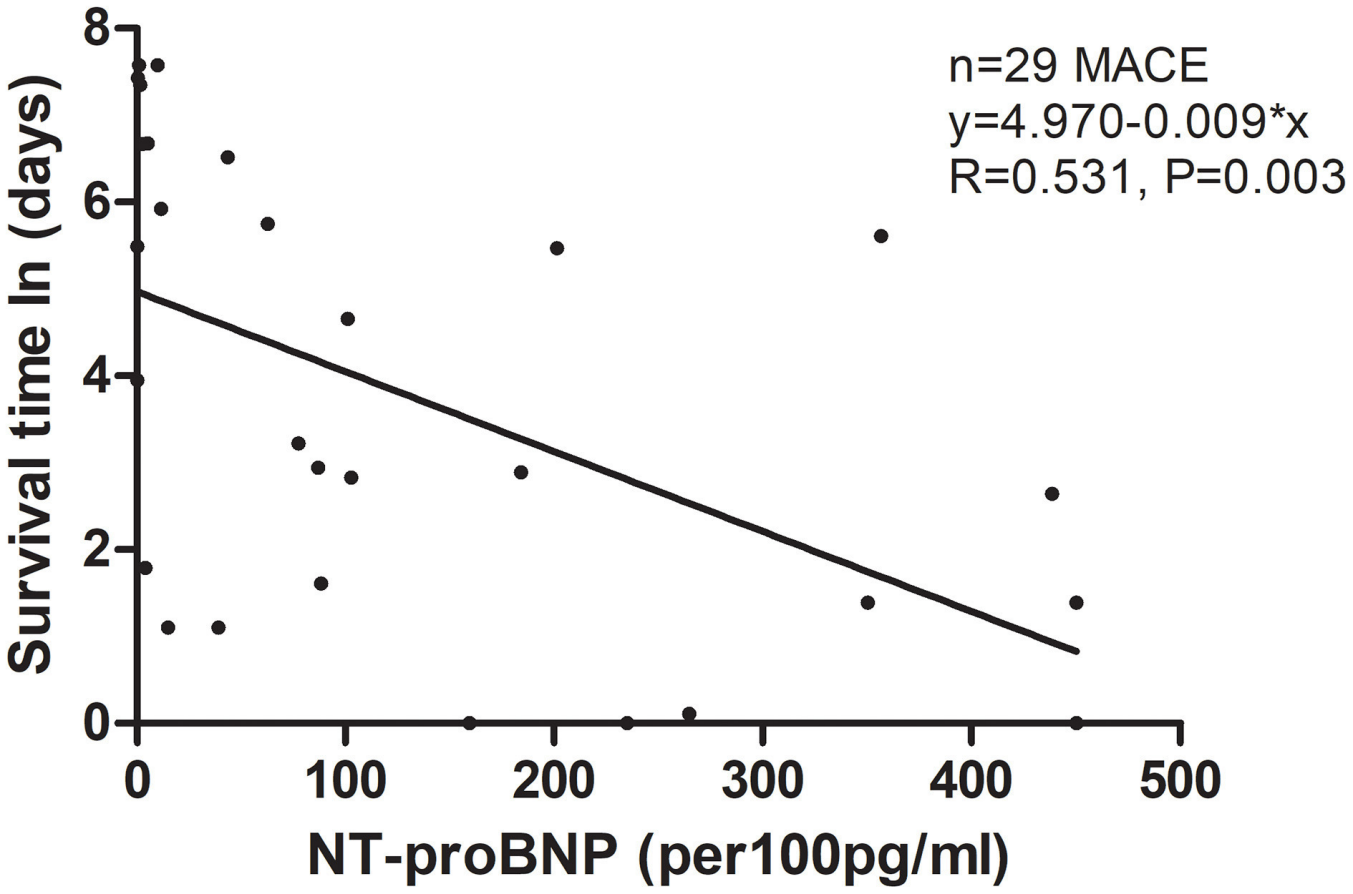
Scatter figure showed a linear correlation between NT-proBNP levels and time of survival (days, Napierian Logarithm scale) in all patients who had long-term MACE (*n* = 29). MACE, major adverse cardiac events; NT-proBNP, N-terminal pro-B-type natriuretic peptide.

Among the four groups, patients in the highest quartile suffered from the highest risk in reaching both 30-day death or heart transplantation and long-term MACE (Figure 3; *P* < 0.001 by log-rank test). The MACE-free survival curve of the patients in the highest quartile was evidently different from those in other quartiles, especially in the first month and the first year. Additionally, Figure 4 shows a negative correlation between NT-proBNP levels and the survival time of the patients who had long-term MACE.

To determine if NT-proBNP was an independent predictor of short-term and long-term adverse outcome, univariate and multivariate Cox analyses were performed (Tables 2, 3). In univariate Cox analysis for long-term MACE, NT-proBNP > 3,549 pg/mL showed a significant predictive value (HR 1.006, 95% CI 1.004–1.008, *P* < 0.001). LVEF (HR 0.936, 95% CI 0.912–0.961, *P* < 0.001), creatinine (HR 1.008, 95% CI 1.005–1.011, *P* < 0.001), age (HR 1.039, 95% CI 1.013–1.066, *P* = 0.003), white blood cell level at admission (HR 1.118, 95% CI 1.030–1.213, *P* = 0.008), right ventricular diameter (HR 1.051, 95% CI 1.008–1.096, *P* = 0.020) and QRS interval (HR 1.011, 95% CI 1.001–1.022, *P* = 0.038) were also predictors of long-term MACE. In multivariate analysis, both of baseline LVEF (HR 0.948, 95% CI 0.919–0.978, *P* = 0.001) and NT-proBNP > 3,549 pg/mL (HR 3.535, 95% CI 1.316–9.499, *P* = 0.012) remained to be strong independent predictors of long-term MACE. For 30-day death or heart transplantation, baseline LVEF (HR 0.919, 95% CI 0.869–0.973, *P* = 0.004), NT-proBNP > 7,204 pg/mL (HR 22.261, 95% CI 1.976–250.723, *P* = 0.012), troponin I level (HR 1.052, 95%CI 1.015–1.090, *P* = 0.005), right ventricular diameter (HR 1.135, 95% CI 1.029–1.252, *P* = 0.011), and age (HR 1.078, 95% CI 1.010–1.151, *P* = 0.024) were independent predictors. Therefore, even after adjustment for other established predictors and confounding factors including LVEF, QRS interval, age, inflammation indicators, and renal function, increased NT-proBNP level was a strong independent predictor for both 30-day and long-term MACE in adult patients with acute myocarditis.

**Table 2:** Univariate and Multivariate Cox Analysis for Long-term MACE.

	**HR**	**95%CI**	***P-*Value**
**Univariate regression**			
Age, y	1.039	1.013–1.066	0.003
Gender, female	1.095	0.508–2.361	0.817
BMI, kg/m^2^	0.937	0.853–1.030	0.180
WBC at admission, × 10^9^/L	1.118	1.030–1.213	0.008
CRP, mg/L	1.002	0.996–1.008	0.593
Creatinine, μmol/L	1.008	1.005–1.011	<0.001
Troponin I, ng/mL	1.014	0.995–1.034	0.147
QRS interval, ms	1.011	1.001–1.022	0.038
QTc interval, ms	1.006	1.000–1.013	0.058
NT–proBNP > 3,549 pg/mL	1.006	1.004–1.008	<0.001
RV, mm	1.051	1.008–1.096	0.020
LVEF at admission (%)	0.936	0.912–0.961	<0.001
**Multivariate regression**			
Age, y	1.024	0.995–1.053	0.113
Gender	0.754	0.301–1.893	0.548
BMI, kg/m^2^	0.926	0.837–1.025	0.138
WBC at admission, × 10^9^/L	1.032	0.925–1.151	0.576
Creatinine,μmol/L	1.003	0.998–1.007	0.238
QRS interval, ms	0.998	0.985–1.011	0.809
RV, mm	1.041	0.984–1.101	0.163
LVEF at admission, %	0.948	0.919–0.978	0.001
NT-proBNP > 3,549 pg/mL	3.535	1.316–9.499	0.012

*Multivariate Cox model selected by a stepwise method with factors that were significant in the univariate analysis. BMI, body mass index; WBC, white blood cell; CRP, C reactive protein; NT-proBNP, N-terminal pro-B-type natriuretic peptide; RV, right ventricular diameter; LVEF, left ventricular ventricle ejection fraction*.

**Table 3:** Univariate and multivariate cox analysis for 30-day death or heart transplantation.

	**HR**	**95%CI**	***P-*Value**
**Univariate regression**			
Age, y	1.052	1.014–1.091	0.006
Gender, female	1.084	0.363–3.236	0.884
BMI, kg/m^2^	0.929	0.811–1.065	0.291
WBC at admission, × 10^9^/L	1.218	1.091–1.359	<0.001
CRP, mg/L	1.004	0.996–1.012	0.366
Creatinine,μmol/L	1.008	1.004–1.012	<0.001
Troponin I, ng/mL	1.027	1.007–1.047	0.007
QRS interval, ms	1.020	1.006–1.034	0.006
QTc interval, ms	1.008	0.998–1.017	0.104
RV, mm	1.079	1.028–1.133	0.002
LVEF at admission, %	0.910	0.875–0.947	<0.001
NT–proBNP > 7,204 pg/mL	33.491	7.480–149.945	<0.001
**Multivariate regression**			
Age, y	1.078	1.010–1.151	0.024
Gender	0.618	0.144–2.661	0.518
BMI, kg/m^2^	0.895	0.748–1.070	0.224
WBC at admission, × 10^9^/L	1.168	0.961–1.419	0.118
Creatinine,μmol/L	1.000	0.993–1.008	0.911
Troponin I, ng/mL	1.052	1.015–1.090	0.005
QRS interval, ms	0.982	0.959–1.006	0.149
RV, mm	1.135	1.029–1.252	0.011
LVEF at admission, %	0.919	0.869–0.973	0.004
NT-proBNP > 7,204 pg/mL	22.261	1.976–250.723	0.012

*Multivariate Cox model selected by a stepwise method with factors that were significant in the univariate analysis. BMI, body mass index; WBC, white blood cell; CRP, C reactive protein; NT-proBNP, N-terminal pro-B-type natriuretic peptide; RV, right ventricular diameter; LVEF, left ventricular ventricle ejection fraction*.

To verify the cut-off value of NT-proBNP abovementioned, Kaplan-Meier survival curves were made and showed that patients with baseline NT-proBNP > 3,549 pg/mL faced higher risk of long-term MACE (Supplementary Figure 1B; *P* < 0.001 by log-rank test), especially in the first year. Similarly, 60% of the patients with baseline NT-proBNP > 7,204 pg/mL died or received heart transplantation within 30 days after admission (Supplementary Figure 1A; *P* < 0.001 by log-rank test).

## Discussion

This study evaluated the predictive value of NT-proBNP in a cohort of adult patients with acute myocarditis. NT-proBNP was a strong predictor for adverse cardiac outcomes both in 30-day and long-term, independently of inflammatory indicators, electrocardiographic and echocardiographic measurements. Among the four quartile groups, patients in the highest quartile of NT-proBNP suffered from not only. In the present cohort, high NT-proBNP level was an independent predictor for adverse cardiac events and provided more information for evaluating the prognosis of adult patients with acute myocarditis. Although NT-proBNP is a well-known marker in predicting poor prognosis in a higher risk of in-hospital death and heart transplantation, but also the poorest long-term outcomes. In the present cohort, high NT-proBNP level was an independent factor associated with MACE and added useful information for assessing the outcomes of acute myocarditis. Although NT-proBNP is a well-known marker in predicting adverse prognosis in different cohorts of cardiac patients, this is the first study evaluating the predictive value of NT-proBNP levels for both of short-term and long-term outcomes of adult patients with acute myocarditis.

NT-proBNP, an inactive N-terminal fragment split from B-Type natriuretic peptide (BNP) prohormone, is released into the circulation by the myocardium due to increased ventricular stress or ischemia (13, 19). Hence it is widely considered a biomarker reflecting the impairment of left ventricular function as well as myocardial ischemia. Previous studies reported that NT-proBNP was a valuable marker of long-term prognostic stratification both in heart failure and coronary heart disease (11–13, 19–21). However, few study assessed its prognostic value in adult patients with acute myocarditis. Previous studies identified that electrocardiographic parameters including pathological Q wave, prolonged QRS duration, and QTc interval were independent predictors for poor outcomes for patients with myocarditis (5–7, 22). Moreover, cardiac dysfunction, LVEF <50%, and right ventricular dysfunction were robust predictors for poor long-term prognosis (23–27). The present study showed that increased NT-proBNP levels were negatively correlated with decreased LVEF, which was widely accepted to be a strong predictor of poor outcomes of acute myocarditis. Moreover, we demonstrated for the first time that baseline NT-proBNP could provide useful information for prognosis after adjusting established factors such as LVEF and QRS duration, suggesting NT-proBNP as a valuable addition to risk stratification for adult patients with acute myocarditis.

The results of our study are in line with the recently published article by Rodriguez-Gonzalez et al. (28) who reported that baseline NT-proBNP > 5,000 pg/mL could help to identify high-risk pediatric myocarditis patients with poor outcomes. BNP was also related to poor prognosis in two smaller studies of pediatric patients with myocarditis (29, 30). In 218 adult patients with acute severe myocarditis, Zhang et al. (31) recently reported that elevated BNP (>100 pg/mL) was an independent predictor for long-term mortality. However, the best cut-off value of NT-proBNP was still uncertain in predicting poor prognosis in the adult patient with acute myocarditis. In the present study, multivariate Cox analysis showed that NT-proBNP was an independent prognostic predictor for both short-term and long-term MACE for adult patients with acute myocarditis. Moreover, according to ROC analysis and evaluated by Cox analysis, NT-proBNP > 7,204 pg/mL and >3,549 pg/mL might be reasonable cutoff values to predict death or heart transplantation within 30 days and to imply long-term MACE, respectively. The cutoff values of NT-proBNP need to be confirmed by a prospective, multicenter study of larger number of patients in the future.

Apart from age, female sex, renal failure, increased NT-proBNP levels are also associated with inflammation (32–34). Previous studies showed that inflammatory cytokines such as interleukin-1β, interleukin-6 (IL-6), and tumor necrosis factor-α could induce natriuretic peptides transcription in cardiomyocytes (35–37). A recently published article by Fish-Trotter also reported that inflammatory conditions were associated with elevated natriuretic peptides release (38). Myocarditis might be caused by various etiology leading to myocardial inflammation and injury. Viral infection, as widely accepted as the most common infectious cause, can harm cardiomyocytes through both direct damage and autoimmune-mediated injury due to systemic inflammatory responses, in which cytokines and antibodies could damage cardiac systolic function and endothelium, leading to systolic dysfunction and ischemia (39). For example, myocarditis is one of the complications in a recent cohort of patients with coronavirus disease 2019 (COVID-19) (40). Guo et al. (14) revealed that NT-pro-BNP elevation was significantly positively linear correlated with myocardial injury in patients with COVID-19. Moreover, Han et al. (16) reported that NT-proBNP was related to the severity and mortality of patients with COVID-19. Thus, in the setting of acute myocarditis, increased NT-proBNP levels might suggest not only cardiac systolic dysfunction, but also acute inflammation and myocardial injury. In this study, we proved that increased NT-proBNP levels were correlated with higher inflammatory factor levels, however, univariate Cox analysis showed no significant association between CRP levels and either 30-day or long-term MACE. But we found that NT-proBNP levels were only slightly correlated with the myocardial injury marker. This might be because of the different extents of myocardial injuries in those patients caused by heterogeneous causes and at different stages of the disease.

Although most patients with acute myocarditis present with mild symptoms and have a good long-term prognosis, a small number of patients with hemodynamically unstable myocarditis, which is called fulminant myocarditis, may have sudden onsets and significant severity with increased in-hospital mortality and poor long-term prognosis (8). The fulminant presentation reflects a more robust immunological and inflammatory response resulting in severe myocyte necrosis and cardiogenic shock (3, 41). Although most of the patients with acute myocarditis have mildly to moderately elevated NT-proBNP levels as a result of inflammation and myocardial injuries, markedly elevated NT-proBNP, which is a marker of severe cardiac dysfunction and extensive myocardial injuries, may help to give useful information for early recognition and prognosis evaluation of patients with fulminant myocarditis. According to the cut-off value by ROC and multivariate Cox analysis, the predictive cut-off value of NT-proBNP for short-term adverse outcomes might be much higher than that of in the diagnosis of heart failure. Hence the measurement of natriuretic peptides may be considered according to the 2020 AHA scientific statement for the recognition and management of fulminant myocarditis (3). Moreover, it is important that the result of our study identifies that NT-proBNP may play a useful role in risk stratification of adult patients with acute myocarditis, independently of other currently used tools including echocardiography and electrocardiogram, and NT-proBNP more than 7,204 pg/mL might be a reasonable cut-off value in acute stage for risk stratification. Patients with a low and moderate elevated NT-proBNP level might need less frequent monitoring and less aggressive treatments. This might lead to possible cost reduction in public health care. Moreover, patients with a high NT-proBNP should be managed more actively and followed up more closely.

Despite the encouraging results, this study has some limitations. First, as a gold standard for diagnosis, endomyocardial biopsy was performed only in 14.1% of these patients and the diagnosis was mainly based on clinical manifestations. Although CMR was performed in 64.1% of the patients, which could compensate for the weakness to a large extent, the possibility of misdiagnosing still existed. Second, due to the follow-up time span of up to 13 years, some patients dropped out, which might lead to a bias to the evaluation of prognosis. There was no significant difference in clinical presentation, laboratory tests and echocardiography parameters between patients with and without follow-up except those lost to follow-up had better baseline cardiac function, which might be the main reason why they did not come back for reexamination. Third, a small number of patients were so critically ill that died before performing an NT-proBNP test, which might introduce selection bias. Last, due to the single-center data and the retrospective design, large-scale and prospective researches are needed to confirm the findings of this study in the future. Nonetheless, our study has several strengths include the complete information of NT-proBNP data, long-term follow-up, and the multivariate analysis adjusted factors that might affect NT-proBNP levels.

## Conclusion

In conclusion, we identified baseline NT-proBNP level as an independent prognostic predictor for acute myocarditis. The NT-proBNP concentration at admission can serve as a valuable biomarker for risk evaluation in adult patients with acute myocarditis.

## Data Availability Statement

The original contributions presented in the study are included in the article/Supplementary Materials, further inquiries can be directed to the corresponding author.

## Ethics Statement

The studies involving human participants were reviewed and approved by Ethics Committees of Fuwai Hospital. The patients/participants provided their written informed consent to participate in this study.

## Author Contributions

YZ designed the study, collected the data, and prepared the manuscript. YZ, NL, and AD performed the statistics. NL and AD provided professional advice on data explanation and revised the manuscript. WZ, HT, and QJ provided professional advice on data interpretation. All authors contributed to the article and approved the submitted version.

## Funding

The work was supported by the Chinese Academy of Medical Sciences Initiative for Innovative Medicine (CAMS-I2M) 2017-I2M-2-002 (to AD).

## Conflict of Interest

The authors declare that the research was conducted in the absence of any commercial or financial relationships that could be construed as a potential conflict of interest.

## Publisher's Note

All claims expressed in this article are solely those of the authors and do not necessarily represent those of their affiliated organizations, or those of the publisher, the editors and the reviewers. Any product that may be evaluated in this article, or claim that may be made by its manufacturer, is not guaranteed or endorsed by the publisher.

## Abbreviations

LVEF: left ventricular ejection fraction
MACE: major adverse cardiac events
HR: hazard ratio
CI: confidence interval
ROC: receiver operating characteristic
AUC: area under the ROC curve.
