# Supplementary material for: Prognosis Implication of N-Terminal Pro-B-Type Natriuretic Peptide in Adult Patients With Acute Myocarditis
Source: Front Cardiovasc Med. 2022 Mar 30;9:839763. doi: 10.3389/fcvm.2022.839763 (PMC9009355; doi:10.3389/fcvm.2022.839763)
Supplement: Supplementary file 1 [file Data_Sheet_1.pdf]

Supplemental material

Table 1. Baseline characteristics of the study population

	All patients (n = 170)
Demographics	
Age (years, Q1-Q3)	31 (24-42)
Male, n (%)	112 (65.9)
BMI (kg/m^2)	24.0±4.1
Comorbidities and NYHA class	
Hypertension, n (%)	13 (7.6)
Diabetes mellitus, n (%)	5 (2.9)
Dyslipidemia, n (%)	15 (8.8)
NYHA III or IV (%)	73 (42.9)
Clinical presentation, n (%)	
Chest pain	61 (35.9)
Dyspnea	70 (41.2)
Syncope	24 (14.1)
Vital signs at admission	
Systolic blood pressure (mmHg)	110±17
Diastolic blood pressure (mmHg)	68±11
Heart rate (beats/minute)	85±22
Electrocardiogram at admission	
QRS interval (ms)	102±30
QTc interval (ms)	442±47
QRS interval >120ms, n (%)	30 (17.6)
QTc interval >440ms, n (%)	78 (45.9)
Arrhythmia, n (%)	
Sinus tachycardia	44 (25.9)
Supraventricular tachycardia	11 (6.5)
Sustained VT/VF	14 (8.2)
complete AVB	24 (14.1)
Bundle-branch block	32 (18.8)
Laboratory tests at admission	
WBC (×10^9/L, Q1-Q3)	7.9 (6.3-11.2)
CRP (mg/L, Q1-Q3)	14.4 (3.8-36.8)
Creatinine (μ mol/L, Q1-Q3)	81.0 (68.7-98.9)
Troponin I (ng/ml, Q1-Q3)	1.9 (0.3-5.7)
NT-proBNP (pg/ml, Q1-Q3)	425.5 (94.4-4557.0)
Peak NT-proBNP (pg/ml, Q1-Q3)	510.8 (125.1-6199.7)
Echocardiography at admission	
LA (mm)	34±6
LVEDD (mm)	49±7
IVS (mm)	9±2

Posterior wall thickness (mm)	9±2
RV (mm)	22±5
LVEF (%)	53±14
LVEF <50%, n (%)	60 (35.3)
Coronary angiography or CT angiography performed, n (%)	126 (74.1)
No evidence of CAD, n (%)	126 (100)
CMR, n (%)	109 (64.1)
T2WI Ratio	2.2±0.8
Late gadolinium enhancement, n (%)	73 (67.0)
Consistent with myocarditis based on Lake-Louise criteria, n (%)	113 (66.4)
Medications	
β-Blockers, n (%)	127 (74.7)
ACE-I or ARB, n (%)	79 (46.5)
Aldosterone antagonists, n (%)	47 (27.6)
Corticosteroids, n (%)	64 (37.6)
Life support treatment	
MCS, n (%)	22 (12.9)
Ventilator, n (%)	15 (8.8)
Temporary pacing, n (%)	20 (11.8)

BMI, Body mass index; VT, ventricular tachycardia; VF, ventricular fibrillation; AVB, atrioventricular block; WBC, white blood cell; CRP, C-reactive protein; NT-proBNP, N-terminal pro-B-type natriuretic peptide; LA, left atrium; LVEDD, left ventricular end-diastolic diameter; IVS, intraventricular septum; RV, right ventricular diameter; LVEF, left ventricular ejection fraction; CAD, coronary atherosclerosis disease; T2WI, T2-weighted images; ACE-I, angiotensin-converting enzyme-inhibitor; ARB, angiotensin receptor blocker; MCS, mechanic circulatory support. Long-term MACE refers to all-cause death, heart transplantation, rehospitalization due to heart failure, documented sustained ventricular arrhythmia (> 30s) and myocarditis relapse.

Table 2. Baseline characteristics of the study population with or without EMB and CMR

	With EMB or CMR (n = 114)	Without EMB or CMR (n=56)	*P* Value
Demographics			
Age (years, Q1-Q3)	32 (24-42)	31 (25-45)	0.954
Male, n (%)	74 (64.9)	38 (67.9)	0.703
BMI (kg/m^2)	23.9±4.2	24.0±3.9	0.864
Comorbidities and NYHA class			
Hypertension, n (%)	10 (8.8)	3 (5.4)	0.549
Diabetes mellitus, n (%)	1 (0.9)	4 (7.1)	0.041
Dyslipidemia, n (%)	10 (8.8)	5 (8.9)	1.000
NYHA III or IV (%)	48 (42.1)	25 (44.6)	0.753
Clinical presentation, n (%)			
Chest pain	43 (37.7)	18 (32.1)	0.476
Dyspnea	54 (47.4)	16 (28.6)	0.019
Syncope	13 (11.4)	11 (19.6)	0.147
Vital signs at admission			
Systolic blood pressure (mmHg)	110±18	111±15	0.908
Diastolic blood pressure (mmHg)	69±11	66±11	0.058
Heart rate (beats/minute)	85±21	86±23	0.748
Electrocardiogram at admission			
Normal, n (%)	29 (25.4)	18 (32.1)	0.358
QRS interval (ms)	98±24	110±38	0.039
QTc interval (ms)	441±43	443±54	0.788
QRS interval >120ms, n (%)	14 (12.3)	16 (28.6)	0.009
QTc interval >440ms, n (%)	52 (45.6)	26 (46.4)	0.920
Arrhythmia, n (%)			
Sinus tachycardia	28 (24.6)	16 (28.6)	0.575
Supraventricular tachycardia	8 (7.0)	3 (5.4)	1.000
Sustained VT/VF	7 (6.1)	7 (12.5)	0.233
complete AVB	10 (8.8)	14 (25.0)	0.004
Bundle-branch block	18 (15.8)	14 (25.0)	0.149
Laboratory tests at admission			
WBC (×10^9/L, Q1-Q3)	7.4 (6.1-10.3)	9.7 (6.9-13.3)	0.021
CRP (mg/L, Q1-Q3)	12.9 (3.8-36.8)	18.7 (5.6-84.3)	0.064
Creatinine (μ mol/L, Q1-Q3)	79.0 (68.7-99.3)	85.2 (68.4-98.2)	0.490
Troponin I (ng/ml, Q1-Q3)	1.34 (0.2-5.7)	3.9 (1.1-8.9)	0.008
NT-proBNP (pg/ml, Q1-Q3)	739.8 (121.9-4656.0)	264.7 (83.5-3431.2)	0.226
Peak NT-proBNP (pg/ml, Q1-Q3)	919.1 (161.5-6278.9)	283.2 (96.5-3599.3)	0.193
Echocardiography at admission			
LA (mm)	34±6	34±6	0.805
LVEDD (mm)	49±7	49±7	0.965

IVS (mm)	9±2	9±2	0.925
Posterior wall thickness (mm)	9±1	9±2	0.188
RV (mm)	22±4	22±6	0.428
LVEF (%)	53±13	53±14	0.934
LVEF <50%, n (%)	41 (36.0)	19 (33.9)	0.794
No evidence of CAD, n (%)	92 (100)	34 (100)	-
Medications			
β-Blockers, n (%)	93 (81.6)	34 (60.7)	0.003
ACE-I or ARB, n (%)	62 (54.4)	17 (30.4)	0.003
Aldosterone antagonists, n (%)	36 (31.6)	11 (19.6)	0.102
Corticosteroids, n (%)	44 (38.6)	20 (35.7)	0.715
Life support treatment			
MCS, n (%)	15 (13.2)	7 (12.5)	0.904
Ventilator, n (%)	7 (6.1)	8 (14.3)	0.090
Temporary pacing, n (%)	7 (6.1)	13 (23.2)	0.001

BMI, Body mass index; VT, ventricular tachycardia; VF, ventricular fibrillation; AVB, atrioventricular block; WBC, white blood cell; CRP, C-reactive protein; NT-proBNP, N-terminal pro-B-type natriuretic peptide; LA, left atrium; LVEDD, left ventricular end-diastolic diameter; IVS, intraventricular septum; RV, right ventricular diameter; LVEF, left ventricular ejection fraction; CAD, coronary atherosclerosis disease; ACE-I, angiotensin-converting enzyme-inhibitor; ARB, angiotensin receptor blocker; MCS, mechanic circulatory support. Long-term MACE refers to all-cause death, heart transplantation, rehospitalization due to heart failure, documented sustained ventricular arrhythmia (> 30s) and myocarditis relapse.

Table 3. Baseline characteristics of the patients with follow-up and lost to follow-up

	With follow-up (n = 151)	Lost to follow-up (n=19)	*P* Value
Demographics			
Age (years, Q1-Q3)	32 (24-42)	30 (24-38)	0.502
Male, n (%)	101 (66.9)	11 (57.9)	0.436
BMI (kg/m^2)	24.1±4.1	22.9±4.4	0.241
Comorbidities and NYHA class			
Hypertension, n (%)	11 (7.3)	2 (10.5)	0.642
Diabetes mellitus, n (%)	5 (3.3)	0 (0.0)	1.000
Dyslipidemia, n (%)	13 (8.6)	2 (10.5)	0.676
NYHA III or IV (%)	69 (45.7)	4 (21.1)	0.041
Clinical presentation, n (%)			
Chest pain	53 (35.1)	8 (42.1)	0.548
Dyspnea	66 (43.7)	4 (21.1)	0.059
Syncope	22 (14.6)	2 (10.5)	1.000
Vital signs at admission			
Systolic blood pressure (mmHg)	110±17	117±16	0.098
Diastolic blood pressure (mmHg)	68±10	71±14	0.301
Heart rate (beats/minute)	86±21	79±24	0.208
Electrocardiogram at admission			
Normal, n (%)	39 (25.8)	8 (42.1)	0.135
QRS interval (ms)	102±30	104±29	0.752
QTc interval (ms)	441±45	443±58	0.880
QRS interval >120ms, n (%)	25 (16.6)	5 (26.3)	0.336
QTc interval >440ms, n (%)	69 (45.7)	9 (47.4)	0.890
Arrhythmia, n (%)			
Sinus tachycardia	39 (25.8)	5 (26.3)	1.000
Supraventricular tachycardia	11 (7.3)	0 (0.0)	0.614
Sustained VT/VF	14 (9.3)	0 (0.0)	0.371
complete AVB	21 (13.9)	3 (15.8)	0.735
Bundle-branch block	29 (19.2)	3 (15.8)	1.000
Laboratory tests at admission			
WBC (×10^9/L, Q1-Q3)	8.1 (6.5-11.4)	6.8 (5.8-11.1)	0.267
CRP (mg/L, Q1-Q3)	14.5 (4.8-53.0)	13.7 (2.6-61.5)	0.504
Creatinine (μmol/L, Q1-Q3)	82.0 (69.0-100.0)	77.0 (63.4-85.8)	0.100
Troponin I (ng/ml, Q1-Q3)	1.9 (0.3-5.7)	1.4 (0.1-8.1)	0.845
NT-proBNP (pg/ml, Q1-Q3)	516.0 (123.0-5024.0)	102.0 (49.3-465.3)	0.005
Peak NT-proBNP (pg/ml, Q1-Q3)	714.0 (157.4-6294.7)	124.7 (53.8-548.2)	0.022
Echocardiography at admission			
LA (mm)	34±6	34±6	0.861
LVEDD (mm)	50±7	47±7	0.130
IVS (mm)	9±2	9±2	0.793
Posterior wall thickness (mm)	9±2	9±1	0.829

RV (mm)	22±5	21±3	0.194
LVEF (%)	52±14	57±10	0.049
LVEF <50%, n (%)	57 (37.7)	3 (15.8)	0.059
Medications			
β-Blockers, n (%)	112 (74.2)	15 (78.9)	0.784
ACE-I or ARB, n (%)	70 (46.4)	9 (47.4)	0.934
Aldosterone antagonists, n (%)	44 (29.1)	3 (15.8)	0.220
Corticosteroids, n (%)	58 (38.4)	6 (31.6)	0.562
Life support treatment			
MCS, n (%)	20 (13.2)	2 (10.5)	1.000
Ventilator, n (%)	14 (9.3)	1 (5.3)	1.000
Temporary pacing, n (%)	18 (11.9)	2 (10.5)	1.000

BMI, Body mass index; VT, ventricular tachycardia; VF, ventricular fibrillation; AVB, atrioventricular block; WBC, white blood cell; CRP, C-reactive protein; NT-proBNP, N-terminal pro-B-type natriuretic peptide; LA, left atrium; LVEDD, left ventricular end-diastolic diameter; IVS, intraventricular septum; RV, right ventricular diameter; LVEF, left ventricular ejection fraction; CAD, coronary atherosclerosis disease; ACE-I, angiotensin-converting enzyme-inhibitor; ARB, angiotensin receptor blocker; MCS, mechanic circulatory support.

Figure Legends

Figure 1. 30-day (**panel A**) and long-term (**panel B**) MACE-free survival in acute myocarditis patients with different baseline NT-proBNP levels. Patients with NT-proBNP > 7204pg/mL died or received heart transplantation more frequently within 30 days than those with NT-proBNP ≤7204pg/mL (*P* <0.001 by log-rank test). Patients with baseline NT-proBNP >3549pg/mL faced higher risk of long-term MACE (*P* < 0.001 by log-rank test), especially in the first year. Long-term MACE included all-cause deaths, heart transplantations, re-hospitalization for heart failure, sustained ventricular arrhythmias (> 30s), and myocarditis relapse.

Figure 2. Receiver operating characteristic (ROC) curve of the ability of NT-proBNP and troponin I to predict 30-day death or heart transplantation (**panel A**) and long-term MACE (**panel B**) in patients with acute myocarditis. In predicting 30-day death or heart transplantation, the area under the curve (AUC) for NT-proBNP was 0.924, with 86.67% sensitivity and 75.48% specificity, while the AUC for troponin was 0.509, with 37.93% sensitivity and 76.60% specificity. In predicting long-term MACE, the AUC for NT-proBNP was 0.733, with 62.07% sensitivity and 80.14% specificity, while the AUC for troponin was 0.616, with 42.86% sensitivity and 93.59% specificity. MACE, major adverse cardiac events; NT-proBNP, N-terminal pro-B-type natriuretic peptide; LVEF, left ventricular ventricle ejection fraction. Long-term MACE included all-cause deaths, heart transplantations, re-hospitalization for heart failure, sustained ventricular arrhythmias (> 30s), and myocarditis relapse.
