# Supplementary figures and images for: Prognosis Implication of N-Terminal Pro-B-Type Natriuretic Peptide in Adult Patients With Acute Myocarditis
Source: Front Cardiovasc Med. 2022 Mar 30;9:839763. doi: 10.3389/fcvm.2022.839763 (PMC9009355; doi:10.3389/fcvm.2022.839763)

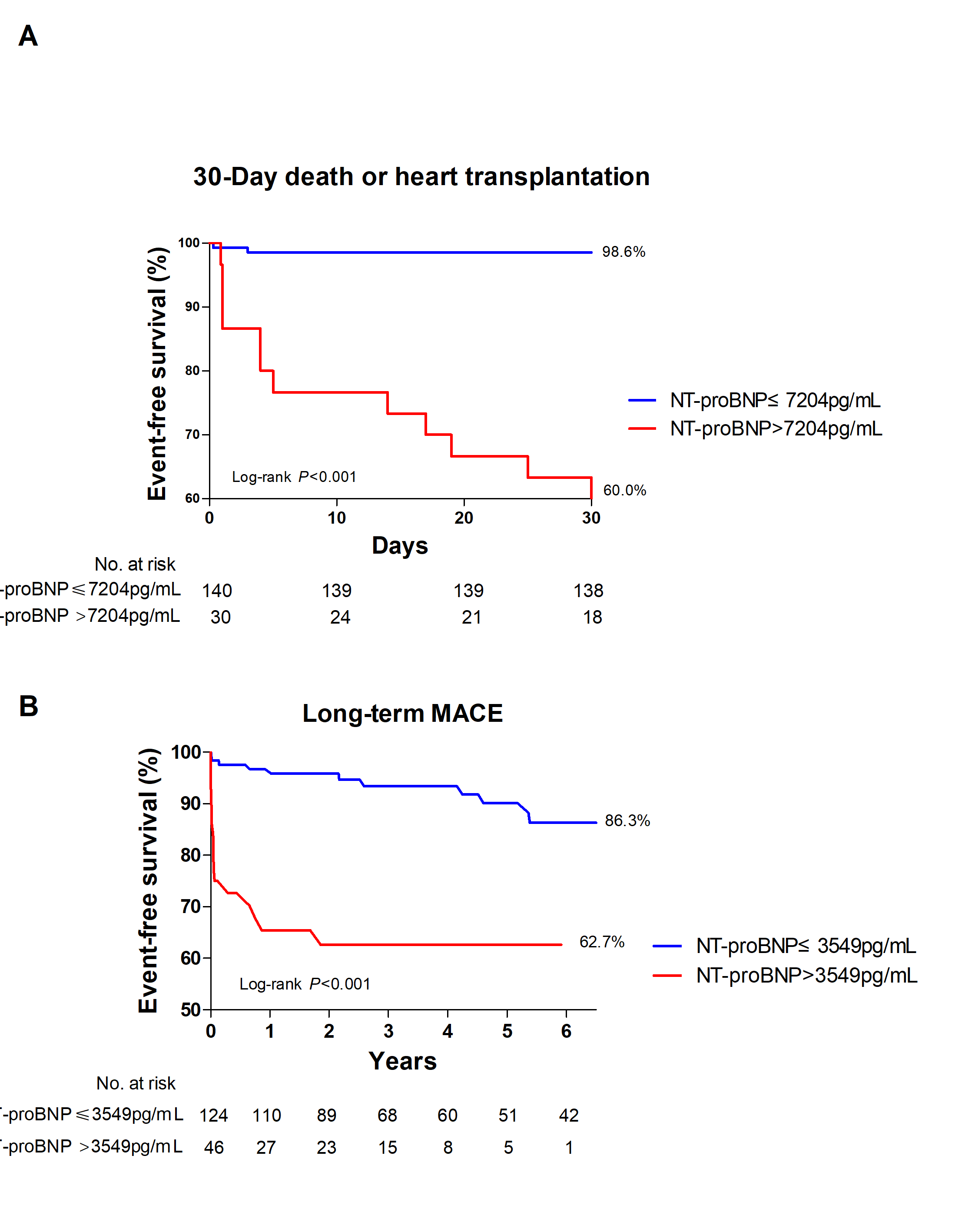

Supplement: Supplementary file 2 [file Image_1.tif]

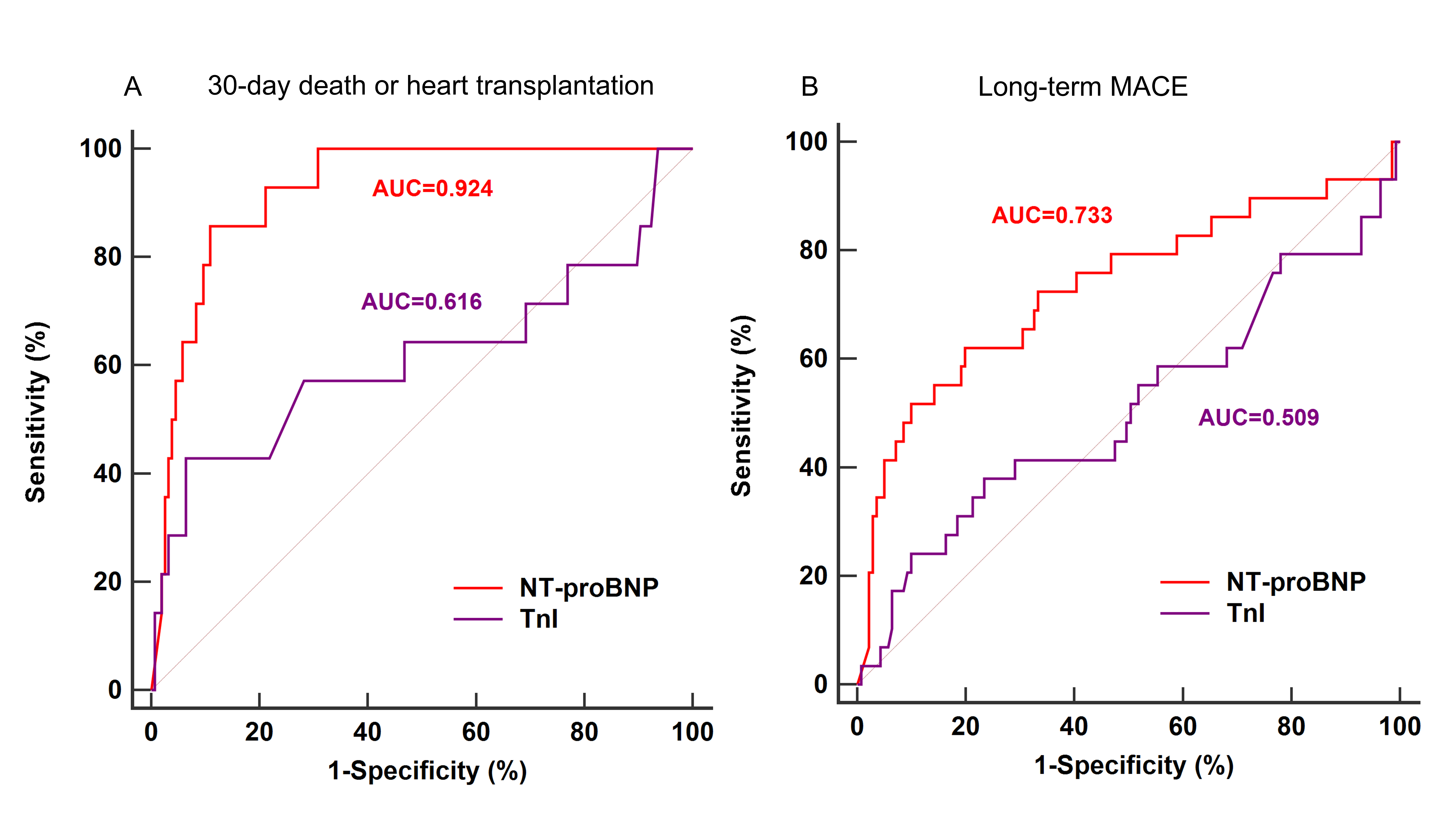

Supplement: Supplementary file 3 [file Image_2.tif]
